# Hot and bothered: Public attitudes towards heat stress and outdoor access for dairy cows

**DOI:** 10.1371/journal.pone.0205352

**Published:** 2018-10-31

**Authors:** Clarissa S. Cardoso, Marina A. G. von Keyserlingk, Maria José Hötzel, Jesse Robbins, Daniel M. Weary

**Affiliations:** 1 Animal Welfare Program, Faculty of Land and Food Systems, The University of British Columbia, Vancouver, Canada; 2 Laboratório de Etologia Aplicada e Bem-Estar Animal, Departamento de Zootecnia e Desenvolvimento Rural, Universidade Federal de Santa Catarina, Florianópolis, Brazil; Tokat Gaziosmanpasa University, TURKEY

## Abstract

On many dairy farms cows are kept indoors. Providing outdoor access is often considered desirable, but housing can protect animals from aversive climatic conditions. For example, by providing shade and fans, indoor housing can protect cows from heat stress they might otherwise experience on open pasture. This study tested how public attitudes to cattle rearing varied when participants were experimentally assigned to different scenarios using a 2 x 2 factorial design varying pasture versus indoor housing with or without heat stress. Participants (n = 581) were randomly assigned to a single scenario, and attitudes in response to the scenario were measured using a Likert scale (1 = “strongly disagree” to 5 = “strongly agree”). We also asked open-ended questions allowing participants to explain their responses. Participants responded most positively to the scenario that provided both pasture access and protection from heat stress (Likert 4.1±0.08), and least positively to scenario with indoor housing and heat stress (Likert 2.2±0.08). However, when the different animal welfare attributes were in conflict (i.e. naturalness as provided by pasture, and biological functioning/affective state as associated with protection from heat stress), participants placed priority on the latter: they were more supportive of the scenario providing indoor housing that protected cows from heat stress (Likert 3.5±0.08), than they were of a pasture rearing system that exposed cows to heat stress (Likert 2.4±0.08). Open-ended responses indicated that participants viewed the lack of protection from heat stress as a failure in the farmer’s duty of care towards the cow. We conclude that participants valued both access to pasture and protection from heat stress for dairy cows, but prioritized protecting animal from heat stress when these features were in conflict.

## Introduction

Previous research has shown that survey participants typically express a preference for systems where cows are able to access pasture [1–3]. Pasture access allows animals to express natural grazing behavior [2], but may also have disadvantages for the cow (reviewed by [4]). For example, outdoor rearing systems often do not provide shelter (e.g. [5]), exposing animals to aversive climatic conditions including excess heat [6,7]. Indoor housing provides some protection from climatic conditions, but can restrict natural behavior [4]. Most dairy farms in the U.S. use indoor housing (~80%); only 7.5% of operations are predominantly pasture-based [8].

Fraser et al. [9] proposed three types of concerns that should be considered when discussing animal welfare: 1) *natural living*–the ability of animals to have natural lives, expressing their natural behavior; 2) *affective states*–related to the capacity of animals to feel well; and 3) *biological functioning*–related to the health of animals. In some situations one type of concern can conflict with another. For example, a cow reared on pasture (arguably good from a natural living perspective) but without adequate shade may feel uncomfortably hot (poor from an affective state perspective) and experience the effects of heat stress (poor from a biological functioning perspective).

It is not clear how people respond to this type of conflicting scenario. One survey found that U.S. consumers consider allowing animals to express their natural behavior outdoors as more important than being at a comfortable temperature [10]. Expression of natural behavior was considered important by Flemish citizens, although not more so than the absence of disease [11]. One recent study used an experimental design to put naturalness and affective state concerns into conflict [12]. Participants were randomly assigned different scenarios describing “Sally” (a chimpanzee) as feeling either very good or very bad, and as living in either a naturalistic or a confined environment. The responses showed that the naturalistic environment especially swayed participants, rating Sally as happier in this setting even when the scenario specified that she was feeling very bad. To our knowledge, no study to date has experimentally contrasted conflicting welfare concerns for farm animals, or indeed examined how people view conflicts between natural living, affective state, and biological functioning concerns.

The aim of this study was to test how public attitudes to cattle rearing vary when experimentally assigned to scenarios that manipulate natural living, affective state and biological functioning concerns. We used a 2 x 2 factorial design varying natural living (pasture versus indoor housing) and affective state/biological functioning (experiencing versus not experiencing heat stress). Based upon the results of Robbins et al. [12], we predicted that participants would express more positive attitudes towards pasture-based rearing even if this was associated with heat stress.

## Methodology

We used an online questionnaire (S1 Questionnaire) developed in Qualtrics (www.qualtrics.com) with Likert scale, open-ended and multiple-choice questions. We used a convenience sample of 601 participants recruited using Amazon Mechanical Turk (MTurk, www.mturk.com). This service provides access to a large pool of U.S. respondents [13], but much fewer from most other countries. To avoid this extra source variation we only included U.S. participants. MTurk respondents are more likely to be young, liberal, urban and single relative to the U.S. population [14]. Although several studies have shown that MTurk provides more representative samples than other types of recruitment (e.g., [13,15]), our sample should not be considered representative and future research may wish to also consider other recruitment services (see [16]).

The study was approved by the University of British Columbia Behavioral Research Ethics Board (H15-03053).

### The questionnaire

Each participant was randomly assigned to one of four hypothetical scenarios using a 2 x 2 experimental design. The two factors were cow housing (pasture *vs*. indoors) and heat mitigation (presence *vs*. absence of shade or fans). The four scenarios were presented as: a–“A herd of dairy cows is kept on pasture where they can graze. The pasture has a shaded area; on warm days the cows are unlikely to suffer from heat stress”; b–“A herd of dairy cows is kept on pasture where they can graze. The pasture has no shaded area; on warm days the cows are likely to suffer from heat stress”; c–“A herd of dairy cows is kept in a barn where they have free access to food. The barn has fans; on warm days the cows are unlikely to suffer from heat stress”; d–“A herd of dairy cows is kept in a barn where they have free access to food. The barn has no fans; on warm days the cows are likely to suffer from heat stress”.

After reading the scenario participants responded to three questions (all on five-point Likert scale) designed to assess their attitude (see [17]) to the scenario: 1) “How much do you disagree/agree with the way these cows are being raised?” (1 = “strongly disagree” to 5 = “strongly agree”); 2) “How inappropriate/appropriate do you consider the cow’s living conditions to be?” (1 = “completely inappropriate” to 5 = “completely appropriate”); and 3) “Do you consider the way these cows are living to be unacceptable/acceptable?” (1 = “totally unacceptable” to 5 = “totally acceptable”). Participants were then asked two open-ended questions: 1) “Please explain your general opinion about the scenario you read”; and 2) “If there was one thing you could change about this farm what would that be?” The number of open-ended questions put to each participant was restricted to these two, as in previous work we have found that the quality of responses declines with the number of questions asked.

Additional questions (also using a Likert five-point scale) assessed potential reasons for the attitude based on an animal welfare construct of affective states (two questions: “How unlikely/likely do you think it is that the cows described in the scenario are suffering?”, 1 = “unlikely” to 5 = “likely”, and “In your point of view how are these cows feeling?”, 1 = “very bad” to 5 = “very good”), biological functioning (one question: “How healthy would you say these cows are?”, 1 = “very unhealthy” to 5 = “very healthy”) and naturalness (one question: “How natural do you consider the environment where these cows are kept?”, 1 = “completely unnatural” to 5 = “completely natural”). We also asked about animal welfare *per se* in two questions: welfare (“How would you describe the welfare of the cows you read about it?”, 1 = “very poor welfare” to 5 = “very good welfare”) and quality of life (“How would you describe the cow’s quality of life?”, 1 = “very bad life” to 5 = “very good life”).

Participants were then asked a series of socio-demographic questions [age, sex, level of educational attainment, residence (urban versus rural)–and income]. In the final section of the questionnaire participants answered four true-false questions to gauge their knowledge about dairy production (all answers were in fact true): 1) “The majority of dairy cows in United States are housed indoors”; 2) “A dairy cow needs to have a calf to keep producing milk”; 3) “The majority of cows and calves are separated from each other within the first few hours of birth”; 4) “Most dairy calves have their horns removed when they are born, either with a hot iron or with a caustic paste”. Responses to these four questions were summed, creating a score that varied from 0 (low knowledge) to 4 (high knowledge).

### Data analysis

#### Quantitative data

For the quantitative analysis we excluded 20 participants who provided invariant responses (i.e. marked the same response on all Likert scale questions) leaving 581 responses for the final analysis (136 in Scenario a; 148 in Scenario b; 145 in Scenario c; and 152 in Scenario d; (S2 Data).

Cronbach’s alpha was used to assess consistency of three first attitude questions (i.e. how much they agreed with the scenario, how appropriate they considered it, and how much they considered the scenario acceptable); the alpha coefficient was 0.95, indicating very good consistency, so these responses were averaged to create a mean for each participant for their *attitude* towards the scenario.

The effects of each of the socio-demographic questions (*age*, *sex*, *education*, *urban/rural* and *income*; 1 df each), *knowledge* of the dairy system (1 df), and the two treatments (i.e. *pasture*, *heat mitigation)* and their interaction (1 df each) on attitude were tested using ANOVA. In preliminary analyses we also tested separately the effect of participant knowledge in relation the *pasture* question (as this was most closely related to the treatments); *pasture* knowledge was not significant, so the final model included only the composite score for knowledge based on all four questions as described above.

To assess how *attitude* related to different components of welfare, we asked respondents questions intended to evaluate their views of the scenario in terms of the cows’ *biological functioning*, *naturalness* of the system, and two questions designed to assess the *affective state* component of welfare. For the latter two questions (*suffering* and *feeling*), Cronbach’s alpha was 0.84 (indicating good agreement), so these two responses were combined to create a mean for *affective state*. Similarly, we asked two questions designed to assess animal welfare, one that specifically referred to *animal welfare* and other phrased as *quality of life*; Cronbach’s alpha was 0.91 (indicating good agreement), so these two responses were averaged to form a construct that we have termed here as *animal well being*. The degree to which *biological functioning*, *naturalness*, *affective state* and the *animal well being* construct were associated with participant *attitude* towards the scenarios was tested using Spearman rank correlations.

Least-square means and standard errors are presented below. Significance was declared for P < 0.05 and a tendency at P < 0.1.

#### Qualitative data

We received a total of 546 qualitative responses to the first open-ended question and 564 qualitative responses to the second open-ended question. These responses were analyzed with the aim of better understanding attitudes towards the scenarios. Responses were analyzed by treatment. Our analysis was based on Minayo [18], using a hermeneutic-dialectic approach that involves exhaustive reading of responses and coding these into themes, giving meaning to the content based on understanding, interpretation and dialectic. To understand the meaning of the content, the coder (the first author) organized the material into topics, with the aim of better understanding responses. The interpretation phase involved re-reading the responses to make sure that the ascribed meanings made sense. The dialectic phase involved re-reading and questioning the interpretations and editing, as needed to improve reliability. For this analysis we focused our approach using the theoretical framework proposed by Fraser et al. [9) that reflects three types of concerns: 1) biological functioning and health; 2) affective states, including pain; and 3) naturalness, including the ability of animals to express natural behavior. We were also open to any other themes that arose following the thematic analyses.

## Results

### Profile of participants

Participant characteristics are summarized in Table 1. By design, all participants were U.S. citizens. Participants were more likely to be younger, male, have greater levels of educational attainment, and somewhat greater income relative to U.S. census averages. Scores for ‘knowledge’ were above that expected by chance, with more than half of participants scoring 3 or 4 out of 4 on the knowledge questions.

**Table 1:** Responses of 581 participants to the socio-demographics questions asked in the survey, presented in relation to U.S. Census Bureau [19–22] averages for the population 18 years and older.

Demographics	Variable	n	%	U.S. Census Bureau (%)
Age (years) ^a^	19–29	220	38	19
	30–39	188	32	18
	40–49	82	14	19
	50 or older	91	16	44
Sex ^a^	Male	324	56	49
	Female	257	44	51
Level of education (25 years and over) ^b^	Less than high school degree	5	1	10
	High school graduate	76	13	29
	Some college but no degree	125	22	16
	Associate degree	95	16	10
	Bachelor’s degree	203	35	21
	Master’s degree	63	11	9
	Doctoral degree	8	1	2
	Professional degree	6	1	1
Area of residence ^c^	Urban	475	82	81
	Rural	106	18	19
Income previous year (US$—household) ^d^	Less than 35,000	203	34	32
	35,000–74,999	238	41	31
	75,000–149,999	122	21	26
	150,000 or more	18	3	11
Dairy knowledge	Four of four questions correct	113	19	n.a.
	Three of four questions correct	191	33	n.a.
	Two of four questions correct	158	27	n.a.
	One of four questions correct	84	14	n.a.
	Zero of four questions correct	35	6	n.a.

^a^ [19)

^b^ [22)

^c^ [20)

^d^ [21)

### Quantitative results

**Fig 1:**
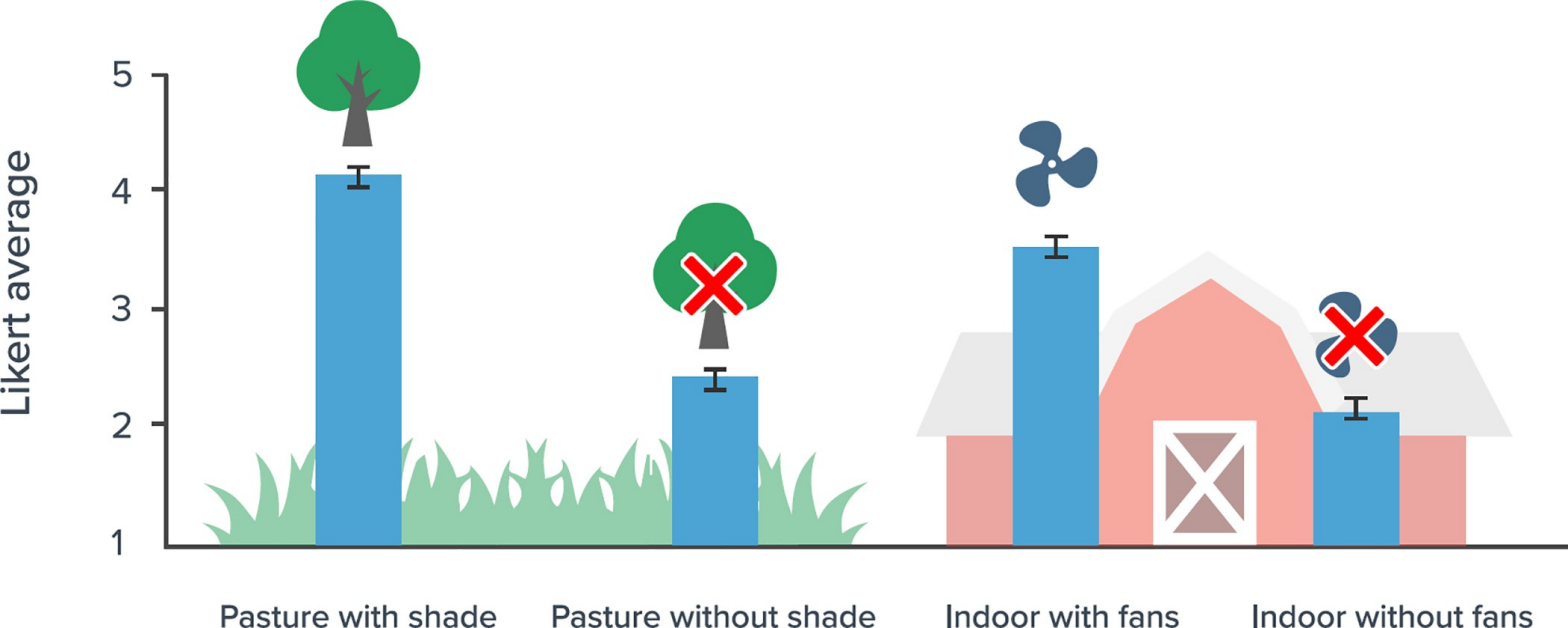
Mean ± SE attitude of participants (n = 581) to scenarios that described dairy cows either having access to pasture or indoor housing, and with or without structures that reduce the risk of heat stress (i.e. shade on pasture and fans indoors). Attitude was a construct consisting of the average of three Likert-scale (1 to 5) questions, where higher numbers indicate a more positive attitude.

Attitudes were more positive to the scenarios with heat mitigation relative to those without (Fig 1; F_1,570_ = 382.8, P<0.0001), and more positive to the scenarios with pasture versus indoor housing (F_1,570_ = 18.4, P<0.0001). We also found evidence of an interaction between these two effects (F_1,570_ = 5.1, P<0.0237), driven by more favorable attitudes to indoor housing with fans than to pasture without shade. Of the socio-demographic and knowledge factors only sex was significant (F_1,535_ = 12.8, P<0.0004), with females having a less favorable attitude than males toward the scenarios (attitude scores averaging 2.9 ± 0.06 *vs*. 3.2 ± 0.05, respectively). We found no interaction between treatment effects and any of the socio-demographic and knowledge factors.

The three individual constructs of animal welfare (naturalness, affective states and biological functioning), and the overall construct of animal well being, were all positively correlated with attitude towards the scenario (Spearman r = 0.55, 0.84, 0.77, 0.87 respectively, P<0.0001).

### Qualitative results

Participants justified their attitude towards the scenarios using arguments related to the cows’ affective state, biological function, and natural living, as well as in relation to a duty to care for the cow and a lack of information in the scenario. These arguments are discussed below.

#### Arguments based on affective states

Participants assigned to the scenario describing cows on pasture with access to shade justified their mostly positive attitudes by arguing that the cows were outdoors, able to graze, with space and shade for warm conditions, such that basic needs were met. For example one participant explained: “I like that the cows have an open pasture to live in and access to enough shade to keep cool on hot days” (P430). Another wrote, “The pasture has a shady area where the cows can go when it’s hot” (P189).

In response to the scenario describing pasture without access to shade, participants described the suffering from heat stress as “inhumane”, and “unacceptable”. For example, one participant explained that “They are living beings and they need shade too…. It’d be nice if they had a shelter” (P98).

In response to the scenario describing indoor housing with fans, positive attitudes regarding were justified on the basis that “[Cows] are being treated in a very humane way” (P78), that “The cows are kept comfortable with fans” (P166). Some specifically stated that some level of confinement was acceptable if this was accompanied with good conditions, “As long as the cows aren’t in pain or very confined, I’m okay with it” (P61), and “If the cows are being treated humanely, I don’t have any issues with them being kept in a barn” (P270). Other participants simply felt that there was no obvious problem with the scenario stating, “There is nothing negative in the cows’ situation” (P138).

Participants in the scenario where cows were living indoors without fans justified their more negative attitudes on the basis that cows may be suffering from heat stress and were not able to go outside, for example stating: “This seems to be animal cruelty” (P234); “The cows don’t have freedom to roam and are trapped in a room. They are subject to uncomfortable heat … that is animal abuse” (P294).

#### Arguments based on biological functioning (health)

Many participants were concerned about cows’ health in scenarios associated with heat stress: “I believe it should be made sure that the cows are as healthy as can be” (P37); “Cows that can find comfortable places to rest are raised as healthier than cows on farms that do not provide such places” (P302); “They are in an unhealthy environment” (P39). Several participants expressed concern that cows might be physically harmed from heat stress, for example: “The conditions in which the cows are raised are not good at all for the health and performance of the cows” (P123); “I believe the cows should not [have] their health jeopardized due to heat stress” (P492).

For the scenario in which fans were provided, people related this to good health. For example, one participant stated “I appreciate that you’ve added fans in the barns for the cow’s comfort/health” (P102).

Participants also felt that heat stress could affect milk quality, e.g. “If they are in too much heat they will be stressed out and it will affect the quality of their milk” (P128), milk production, e.g. “I suspect the fans ensure the cows are comfortable enough to produce more milk” (P102), and reproduction, e.g. “If the cows are experiencing heatstroke, that means they’re not healthy. If they’re not healthy, they’re probably going to have problems reproducing” (P569).

A few participants expressed concern about cows dying from the heat stress. For example, one claimed that “The cows are certain to die under these conditions” (P328) and another stated “Dying of heat exhaustion would be a cruel and painful way to die” (P451).

#### Arguments based on naturalness

Some participants expressed positive attitudes towards the scenario describing pasture access without shade, often basing their arguments on naturalness. For example, one participant stated that “They are cows, being outside is their natural environment” (P134), and another argued that, “I think that it would be preferable if the cows had shade. However, it’s much better that they are on pasture versus living in a barn or in confined corrals between milkings” (P464). A few seemed to implicitly agree with the welfare trade off stating, “I think it is perfectly fine given the information” (P361). Others were more explicit about the acceptability of the trade off arguing, “I don’t like the fact that the animals can suffer from heat exposure. However, I believe it is better than suffering by being constantly locked up” (P459).

Similarly, the negative attitudes of some participants to the scenario describing indoor housing with fans were sometimes based upon concerns about naturalness. One participant wrote, “I think that cows should be able to be outside and not stuck in a barn” (P70). Another claimed “Cows are animals and shouldn’t be caged, even in a barn. They should be allowed to free roam and enjoy the sunshine and grass!” (P306). Several explicitly argued that having fans within the barn was not enough to justify the lack of pasture access. For example, one stated “So, because a dairy cow has access to food and gets a fan to circulate the air this is supposed to be considered humane? (…) These cows are not properly cared for” (P256). Yet another argued “It’s nice that the cows are being kept in a comfortable living area, but it’s only natural and fair for them to be allowed to go outside sometimes” (P332). A third argued “[Although] conditions are relatively comfortable, it’s not the natural way of things in my opinion” (P572).

Some participants responding to the pasture without shade scenario seemed to justify the practice by comparing this to naturalistic situations. For example “Cows are made to graze in the sun. They are animals” (P396), and “That is probably how many cows go through life. The fact is that most cows probably experience much, much, worse circumstances. In the ‘wild’ they would probably also experience heat stress without man made shelters and assistance” (P285). Others used comparisons to cattle living in what they considered standard conditions on farms stating, “It is not ideal but better than most commercial operations” (P341), and “Cattle don’t have shelters while roaming on large pastures that cover thousands of acres. They have survived for years without shelter” (P584). Similarly, participants responding to the scenario describing indoor housing without fans often used comparisons to natural conditions, stating “Cows in the wild wouldn’t have fans either so these cows don’t necessarily need fans either” (P174), “I don’t think that in nature animals get fans” (P321), and “Cows in nature don’t have air conditioning” (P350).

#### Arguments based on a duty of care

Some participants argued that it was a moral responsibility to provide protection to the cows stating, “The cows are providing [the farmers] with an income, the least they could do is give them a shaded area” (P221). Participants often expressed some moral judgment about the way farmers were rearing their cows. For example, participants commented that they could not understand why farmers would not plant trees, a solution that they believed was obvious and easy to apply. For example, “I don’t really see any excuse for them to be in the sun all day long with no shade” (P183), “There’s no excuse not to provide any type of shade under these conditions” (P473), and “Shelter would not be that hard or expensive to construct” (P496). Similarly, they felt that fans (for indoor housing) were easy and inexpensive to install stating, “Since it is easily fixable, it is cruel and inhumane to make them suffer now” (P54), “I’m not sure why cows can’t get fans installed …? It doesn’t make any sense” (P191), and “The cows are suffering needlessly from a problem that seems to have an inexpensive solution (fan blowing)” (P429).

#### Arguments based on a lack of information

Several participants expressed a desire for more information. For example, in response to the pasture with shade scenario participants stated “I need more details. It does sound like a nicer condition than most cows are raised in, but I only see the positives here. What other conditions like how they’re fed/etc. are in play?” (P284), and “I didn’t receive enough information to be able to make an informed judgment about the cows’ living conditions” (P413). For the pasture without shade scenario one participant said, “I would need to see the environment first to understand what is really happening” (P487). Similarly, participants assigned to the indoor housing with fans scenario argued “My answers were neutral because I felt I wasn’t given enough information about the cows’ conditions within the space, other than temperature” (P525), and “I felt there wasn’t enough information to form an opinion on how the cows were being raised/treated. How much space do they have to walk around? Are they allowed to go outside?” (P91). Some participants in the scenario that was most negatively evaluated by participants (indoors without fans) made similar arguments, stating “I don’t have enough information on this matter, I don’t know if they must be kept this way for the better of the dairy” (P305), and “I don’t have a strong opinion about this. I’m not sure how much animals feel pain and suffer, so I don’t have a strong answer” (P472).

#### Improving the environment–changing scenarios

**Fig 2:**
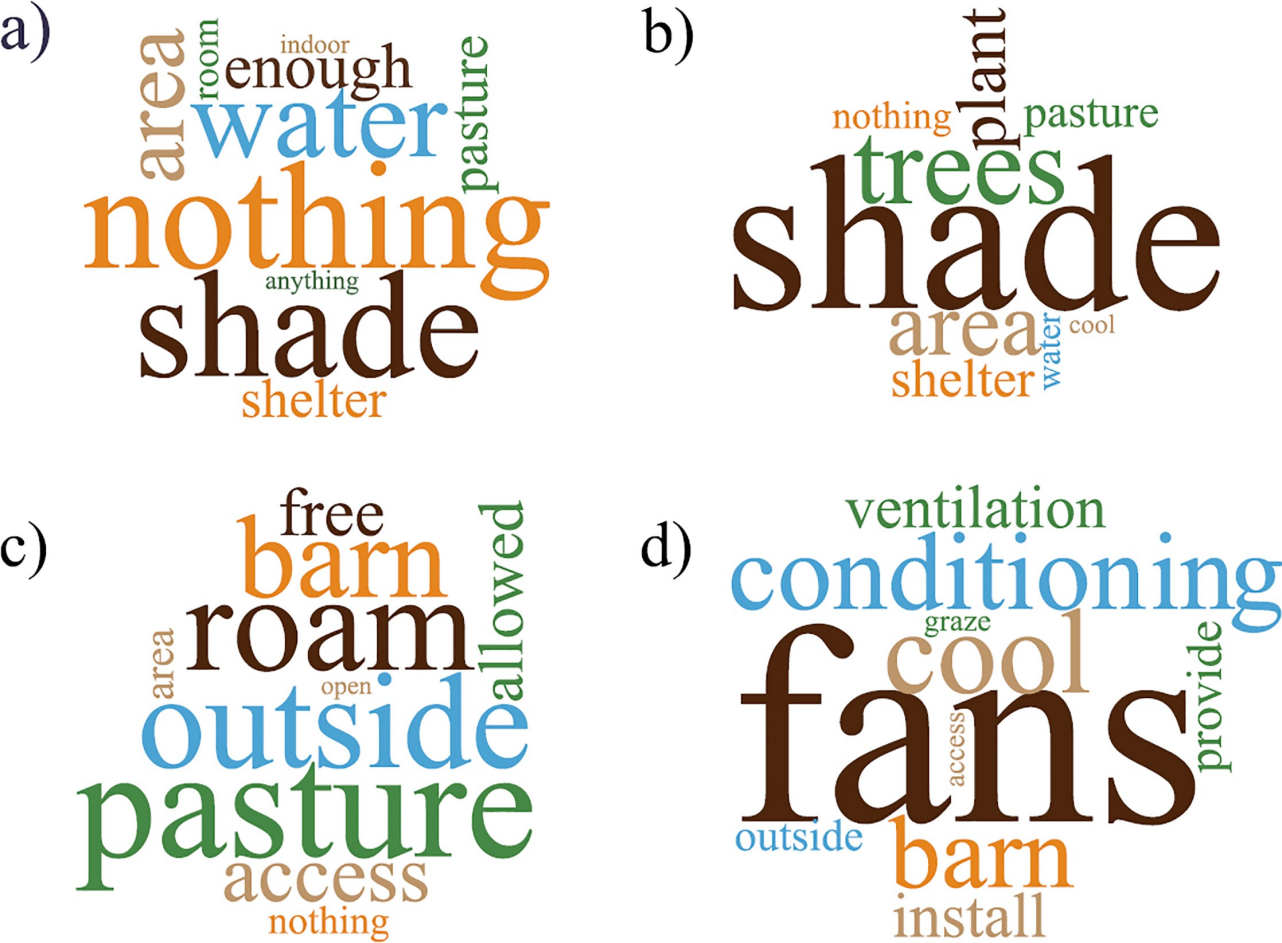
Word clouds generated using the 10 most frequently used words in response to the question “If there were one thing you could change about this farm what would that be?” The words appearing in larger type were used most frequently. Responses of participants assigned to the four scenarios are shown separately: a–pasture with shade (n = 128); b–pasture without shade (n = 144); c–indoor housing with fans (n = 143); and, d–indoor housing without fans (n = 149).

To better identify what participants considered problematic, they were asked to suggest changes to the scenario. People assigned to the scenario with pasture and shade suggested improvements in the amount of resources available to the animals (e.g. more shade, water, and space) and stated that is was important that cows were healthy in that environment. For the scenario with pasture but without shade, participant comments focused on planting trees for shade and constructing some shelter to protect the cows (Fig 2).

Participants assigned to the indoor scenarios often suggested allowing the animals to go outside, including providing cows access to pasture with trees for shade. This hybrid solution was considered to provide conditions described as “pretty much ideal”, a “good place to live”, and a “very humane situation”. One quote summarized this desired scenario: “The best scenario would be to let the cows freely graze on pasture which is shaded with trees, and when they are in the barn to be fed or milked, have an evaporative cooling system in operation” (P148).

## Discussion

The results of this study indicate that participants valued both access to pasture and protection from heat stress when assessing rearing conditions for dairy cattle. In addition, when participants were given a scenario in which cows were kept indoors, but provided fans to mitigate heat stress, they showed a more positive attitude than did participants given a scenario in which cows were kept on pasture, but without access to shade. In this situation at least, it seems that participants prioritized welfare concerns about heat stress (associated with poor affective states and biological functioning) over concerns about access to the outdoors (associated with naturalness). Thus, the most favorable attitudes were to the option of pasture with shade, followed by indoor housing with fans; the other two options (outdoors with no shade and indoors with no fans) were perceived much less favorably. We included quantitative assessments of the scenarios in terms of different welfare components (naturalness, affective state and biological function), as well as for an animal welfare construct we termed well being [23]. We found that all welfare components and the overall construct well being were highly correlated with the participants’ attitude towards the scenario, especially so for biological functioning and affective state. The increased weighting of biological functioning and affective state over naturalness may explain why participants favored the scenario without heat stress versus that without pasture access when these features were in conflict.

Previous work has suggested that affective-state concerns are most important in assessments of animal welfare [24], but other work has suggested that naturalness is the most important characteristic, particularly for people not involved with animal production [10,11]. Our data suggest that failure to provide protection from heat stress was considered unacceptable; participants described scenarios without heat mitigation as “inhumane” and “unacceptable”. Also, a number of participants specifically commented on the obligation of farmers to protect their cows, and viewed the lack of heat mitigation as an unacceptable breach of this obligation. Our study suggests that although participants preferred that cows have access to the outdoors, they would not support systems that failed to provide protection from heat stress.

Arguments based on naturalness were cited in the open-ended responses to justify attitudes. People often associate naturalness with organic production, health and the environment [25,26]. Previous work in different cultural and geographic contexts has shown that naturalness is often perceived as important (reviewed by [27]), perhaps especially in the context of food [28]. A series of studies in the U.S. and Europe have illustrated some of the meanings of naturalness and reasons for people’s positive attitudes towards this characteristic. Rozin et al. [29] argued that people’s preference for natural is explained by the latent belief that “natural is inherently better, in moral and/or aesthetic senses”. In a recent review about the importance of naturalness in food, Román et al. [28] categorized three attributes of naturalness that are important for people: 1) organic or local; 2) ingredients (e.g. preservatives, chemicals, hormones, pesticides and GMOs), and processes (e.g. traditional or homemade); and 3) healthy, eco-friendly, tasty and fresh. Rozin et al. [26] found that people relate natural much more with plants than with animals, but often cite characteristics related to naturalness in farm animal contexts (e.g., [2,3]). Naturalness may be more important when considered in relation to animal products (such as milk), and less important when animals themselves are the focus (as in our study). We encourage future research to better understand why people view naturalness as important in farm animal contexts.

Participants may have experienced some degree of cognitive dissonance [30] when confronting these scenarios. Perhaps as an attempt to avoid this conflict some participants made comparisons between the scenario and either what was natural or common on dairy farms (thus arguing that the situation was not that bad). Others suggested that installing shade and cooling systems was cheap and straightforward (thus suggesting that the solutions to the dilemma were easy), and that we did not provide enough information (thus allowing them to abstain from judgment).

One limitation of this study is that we provided relatively little detail for each of the scenarios. For example, instead of providing a detailed description of a specific indoor housing system, we simply referred to this as a “barn”. Specific characteristics of indoor housing systems might have affected the attitudes; for example, we expect that attitudes would have been more negative had we specified tie stall housing (in which animals are tethered into a specific stall) versus free stalls (in which cows are free to roam around the barn). We also presented the treatments as binary conditions, even though heat stress is likely to vary in a more continuous fashion. In addition, the heat mitigation methods specified in the two housing treatments might have been perceived to have different effects, as the barn treatment provided both shade (from the barn roof) and airflow (from the fans), while the pasture condition provided only shade (air flow was not controlled). Thus even though we stated in the scenarios that the cows provided heat mitigation both in the barn and on pasture were ‘unlikely’ to experience heat stress, astute readers may have surmised that the heat mitigation was more effective in the barn. Readers with more experience in dairy barns may have also assumed that the “fan” treatment also included some type of water spray as these treatments are often combined.

Although some participants believed that planting trees for shade is straightforward, research suggests that farmer’ attitudes to trees on pasture can be complex [31]. Access to high quality plant material, management skills, information, technical advice and damage by cattle, leaf-cutting ants, wind or competition by grasses can be barriers for farmers [32,33]. We suggest that a failure to plant trees should not be viewed as a simple case of neglect, and encourage new research examining the perceived social, economic and technical barriers faced by farmers. This issue highlights the distance between rural and urban citizens (see [34]). For this issue it may be helpful to create forums for conversation about contentious issues, allowing a better understanding of citizens’ views and farmers’ constraints [35].

Other than sex, socio-demographic characteristics did not relate to participant attitude towards the scenarios. Sex effects are commonly reported in studies on attitudes towards animal welfare, with females on average being more concerned than males [26]. The lack of other demographic effects suggests that the concerns we have identified are broadly held across a range of demographic categories. This generality may be associated with familiarity of the issue, and that our participants had high scores in basic knowledge about dairy production. For example, some participants correctly reported that cows could die from heat stress [36,37]. Mistreatment has been reported on U.S. dairy farms in recent years [38–40], so this issue was likely also familiar to participants [41–43].

Previous studies have reported that MTurk participants tend to be younger and more urban than expected in a representative sample of U.S. participants [14]. Participants in the current study were younger, but not more urban compared to census averages. Our study had more male participants, with greater levels of education and income than the general population [19,21,22]. Moreover, the study design required that all participants had Internet access. We remind readers that we found no association between socio-demographic characteristics and attitudes to the scenarios.

Most respondents correctly responded that most dairy cows in U.S. were kept indoors. Knowledge about farming can ameliorate some attitudes [44] and can contribute to forming more complex opinions about farming [2]. The results might differ for participants from countries where most of cows are pasture reared (e.g., Brazil and New Zealand).

When asked what changes participants would like in the scenario, they often called for mixed systems (providing access to well managed indoor and outdoor spaces). Other studies have found that these systems can work well for cows, allowing them to choose to enter the barn or to visit pasture depending upon the time of day, season, where feed is provided and previous experience on pasture [4]. We did not provide a hybrid scenario but predict that attitudes would be favorable to this option.

## Conclusion

Attitudes of a convenience sample of U.S. citizens were most favorable to rearing systems that included pasture and shade, and least positive to indoor systems without fans; however, attitudes were more favorable to indoor housing with fans than to pasture without shade. On the basis of these results, and those of our qualitative analysis, we conclude that participants highly value both thermal comfort and pasture access, and that one type of animal welfare concern (e.g. the naturalness of pasture) does not trump others (e.g. affective state and biological functioning concerns associated with heat stress).

## Supporting information

S1 Questionnaire (DOCX)

S1 Data (CSV)
