# Supplementary material for: Hot and bothered: Public attitudes towards heat stress and outdoor access for dairy cows
Source: PLoS One. 2018 Oct 31;13(10):e0205352. doi: 10.1371/journal.pone.0205352 (PMC6209170; doi:10.1371/journal.pone.0205352)
Supplement: S1 Questionnaire — (DOCX) [file pone.0205352.s001.docx]

## Questionnaire “Hot and bothered” – PONE-D-18-12899

**Read carefully the following scenario**

Scenario A – A herd of dairy cows is kept on pasture where they can graze. The pasture has a shaded area; on warm days the cows are unlikely to suffer from heat stress.

Scenario B – A herd of dairy cows is kept on pasture where they can graze. The pasture has no shaded area; on warm days the cows are likely to suffer from heat stress.

Scenario C – A herd of dairy cows is kept in a barn where they have free access to food. The barn has fans; on warm days the cows are unlikely to suffer from heat stress.

Scenario D - A herd of dairy cows is kept in a barn where they have free access to food. The barn has no fans; on warm days the cows are likely to suffer from heat stress.

**Now please answer the follow questions about the scenario above**

1. How much do you disagree/agree with the way these cows are being raised?

strongly disagree (1) (2) (3) (4) (5) strongly agree

1. How inappropriate/appropriate do you consider the cow’s living conditions to be?

completely inappropriate (1) (2) (3) (4) (5) completely appropriate

1. Do you consider the way these cows are living to be unacceptable/acceptable?

totally unacceptable (1) (2) (3) (4) (5) totally acceptable

**Please explain your general opinion about the scenario you read. (open-ended question)**

_________________________________________________________________________________________________________________________________________________________________________________________________________________________________________________________________________________________________________________________________________________________

**If there were one thing you could change about this farm what would that be? (open-ended question)**

_________________________________________________________________________________________________________________________________________________________________________________________________________________________________________________________________________________________________________________________________________________________

1. How unlikely/likely do you think it is that the cows described in the scenario are suffering?

unlikely (1) (2) (3) (4) (5) likely

1. In your point of view how are these cows feeling?

very bad (1) (2) (3) (4) (5) very good

1. How do you describe the welfare of the cows you read about it? (Considering that animal welfare includes health, feelings and naturalness)

very poor welfare (1) (2) (3) (4) (5) very good welfare

1. How would you describe the cow’s quality of life?

very good life (1) (2) (3) (4) (5) very bad life

1. How natural do you consider the environment where these cows are kept?

completely unnatural (1) (2) (3) (4) (5) completely natural

1. How healthy would you say these cows are?

very unhealthy (1) (2) (3) (4) (5) very healthy

**Socio-demographic questions**

Age (years)

Sex (Male/Female)

Which of the following best describes the area where you have lived most of your life? (Urban / Suburban / Rural)

Education

Income

**Finally, please respond “false” or “true” for the follow affirmatives:**

1. The majority of dairy production in United States are housed indoors (False/True)
2. A dairy cow needs to have a calf to keep producing milk (False/True)
3. The majority of cows and calves are separated from each other within the first few hours of birth (False/True)
4. Most dairy calves have their horns removed when they are born, either with a hot iron or with a caustic paste (False/True)
